# The impact of high-intensity arm crank exercise on reaction time in wheelchair fencers: gender differences and mechanical predictors

**DOI:** 10.1038/s41598-024-62013-2

**Published:** 2024-05-27

**Authors:** Michal Starczewski, Patrycja Bobowik, Piotr Kazimierz Urbanski, Stefan Makowski, Michal Morys

**Affiliations:** 1 Faculty of Rehabilitation, Józef Piłsudski University of Physical Education in Warsaw, Warsaw, Poland; 2 Department of Adapted Physical Activity, Poznan University of Physical Education, Poznan, Poland; 3 Szermierka Wolomin, Wolomin, Poland; 4 The Jerzy Kukuczka Academy of Physical Education in Katowice, Katowice, Poland

**Keywords:** Human behaviour, Physiology

## Abstract

To achieve high performance, wheelchair fencing (WF) athletes are required to exhibit good physiological and timing indicators. The main aims of this study were to assess the relationship between the results of the repeated sprint ability (RSA) test and reaction time (RT) in WF, and to evaluate changes in RT after repeated high-intensity sprints in the group of an international-level WF athletes. This experimental study involved 18 athletes (aged 34.6 ± 7.70) from the Paralympic WF team. To establish the impact of fatigue on psychomotor capacity, the participants undergo a series of tests. At the beginning of the study, first reaction time (RT1) was measured. Afterwards, the RSA test was performed using the arm crank ergometer to evaluate the participants’ repeated sprint ability. Immediately after RSA, the second reaction time (RT2) was measured. Statistical analysis revealed moderate correlations between the RT2 and total work, decrease of work (DW), highest peak power, mean peak power, and highest peak power/kg, but these correlations were not statistically significant (p > 0.05). All fencers achieved a significantly shorter average RT2 (p < 0.005) after the RSA test (0.383 ± 0.035 s) than before the test (0.391 ± 0.038 s). Additionally, RT2 was significantly shorter than RT1 in the women's group (p < 0.001). Moreover, males had significantly greater values of repeated sprint ability parameters: highest work, total work, decrease of work and highest peak power (p < 0.05) than females. To conclude, repeated high-intensity arm crank exercise has a positive impact on simple postexercise cognitive tasks in WF fencers, especially in women, and leads to a decrease in RT. The RSA parameters can be predictors of changes in RT in men and women wheelchair fencers.

## Introduction

Wheelchair fencing (WF) is a Paralympic sport that is practiced by athletes with physical disabilities that has been featured at every Paralympic Games since its commencement^1^. WF is also defined as a sport in which psychomotor and coordination-related abilities need to be harmonized with strength and explosiveness as exercise capacity^2^. To achieve high results, fencers must be characterized by high concentration, quick response to a stimulus (reaction time—RT), short movement time (MT), developed and automated movement patterns^2^.

During the implementation of the training process and while striving for sports mastery, appropriately selected training tasks are used to develop the abovementioned features^3^. A study conducted with 16 international WF coaches showed that increased athletic success was also associated with qualities of strength, power, flexibility, and motor control of the trunk and fencing arm^1^. During the fight, utilize the movement of their torso in conjunction with an arm holding the weapon, which determines the fencers’ range of attack in order to earn points^2^. This, in turn, depends on the intensity of the fight, will force them to generate maximal values of strength and power^1^. To perform high-intensive movements in a short time to attack the opponent or to defend will result in an increase in the use of anaerobic energy sources, mostly from the breakdown of phosphocreatine to rebuild adenosine triphosphate^4^. Previous studies have not shown that the power generated from anaerobic sources affects RT and performance in WF but presented an analysis of aerobic capacity and its influence on restitution and thermoregulation during WF fight^5,6^.

A round of wheelchair fencing lasts three minutes, during which competitors fight to score? 5 hits. Individual actions are divided into repositioning in a fighting position^7^. Intermittent exercises of short duration involve the use of alactic energy sources to minimize MT. Generating high movement speed and thus power in a short time significantly affects the outcome of single bouts^1^. These statements were investigated in Olympic fencing. Turner et al. stated that strength and power qualities are essential for athletes in this discipline. Moreover, they recommended high-intensity interval training to reduce the accumulation of blood lactate, which suggests that fencing anaerobic energy sources play a crucial role^8^. In previous studies, the problems of generating power and maintaining power during repeated efforts have not been studied among WF. However, determining changes in anaerobic capacity during short-term, repeated efforts could help identify parameters that can be determinants of sports success in WF as in other disciplines^9,10^.

In fencing, it is extremely important to react quickly to the opponent's activity, which may be disturbed by fatigue resulting from the stage of the tournament, the length of the fight or external conditions^8^. Moreover, the authors of a previous study claimed that RT plays a key role in fencing performance^3^. This finding was also confirmed by the opinions of experienced national team coaches. Eighty percent of them agreed that RT is one of the key features associated with obtaining the most favorable results in WF^1^. Strength and conditioning based on exercise focused on improving motor skills and RT between an unexpected stimulus and the response to it are the basic ways to assess motor quality^11–13^. Considering the use of different drills during warm-up protocols, we need to remember that they can affect RT and appropriate workloads or that restitution methods can be crucial for maintaining RT during the final fights of a tournament^14^.

The use of conditioning in the form of repeated sprints has a positive effect on changes in RT in students^15^. Previous studies indicate that sports training has a positive impact on RT and that the development of repeated sprint abilities (RSA) results in athletes without disabilities^15,16^. The combination of these two types of training suggested that there should be a relationship between changes in RT and mechanical indices values of RSA test performed on the upper limbs in WFs; however, these changes have not been widely described in the literature thus far. Therefore, the aim of this study was to assess the relation between the repeated sprint ability parameters obtained in the RSA test and RT in WF. The second aim was to assess changes in RT after repeated high-intensity arm crank exercise in the group of professional WF athletes.

## Materials and methods

### Subjects

Ethical clearance for the study was granted by the Józef Piłsudski University of Physical Education in Warsaw Ethics Committee (SKE 01-55/2022) and all research was performed in accordance with the Declaration of Helsinki. Before taking part in the research, the written informed consent form to take part in the study and to publish images was signed by each participant. A power analysis revealed that the study design could reliably detect correlation whit moderate effect size = 0.6 with a probability > 0.8, assuming a two-sided criterion allowing for a maximum Type I error rate of α = 0.05. The study involved 18 athletes aged 34.6 ± 7.70 (females: n = 6; aged 34.3 ± 7.20; males: n = 12; aged 34.7 ± 8.25) from the Paralympic wheelchair fencing team. The testing sessions took place at an ambient temperature ranging from 18 to 22 degrees Celsius. All fencers were tested between 10:00 a.m. and 1:00 p.m. to ensure consistency of the measurements. Prior to the testing, fencers were instructed not to engage in intense exercise on the day before and to have their last meal at least 2 h before the scheduled test time.

Athlete body weight was measured on a wheelchair scale (males: 80.2 ± 6.48 kg; females: 66.2 ± 14.40 kg; both: 75.5 ± 11.6 kg). All the participants had at least 2 years of experience in international competition. Moreover, among participants were multiple Paralympic, World Championships and World Cup medalists.

### Study protocol

**Figure 1:**
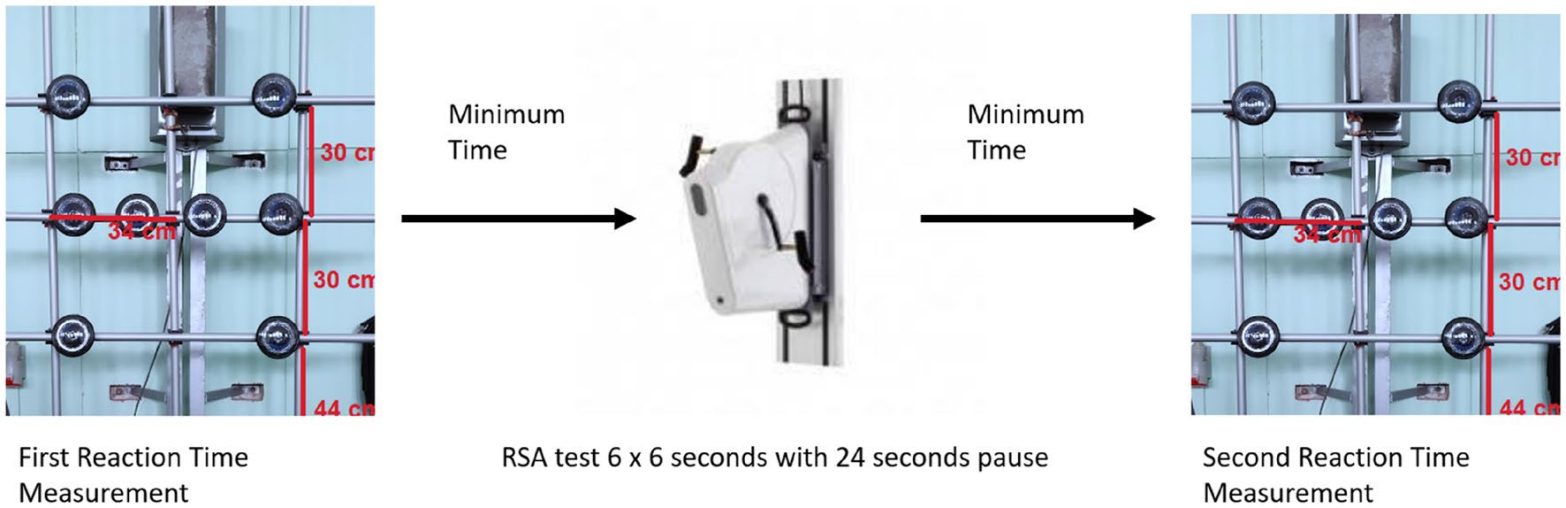
Study protocol scheme.

After the anthropometric measurements were taken, each participant completed a familiarisation to the tests and, as a next step, reaction time (RT1) measurement to establish the resting RT. Afterwards, a repeated sprint ability (RSA) test, which mimicked 3 min of WF fight was performed. Immediately after the end of the RSA, a second reaction time (RT2) measurement was performed (Fig. 1).

### Repeated Sprint Ability Test (RSA)

The RSA test was conducted using the LODE Angio arm crank ergometer to evaluate the participants’ repeated sprint ability. The test consisted of six intervals lasting 6 s each with maximal effort, followed by 24 s of passive rest. The duration of the test was adapted to the duration of the 3-min WF round during the competition. Fencers were instructed to arm crank at the highest possible cadence while bearing weight during each interval. Friction weight varied from 2 to 6% of body mass and was established during pretests individually for each athlete, depending on sex and functional capacity. Before the test, there was a 5-min warm-up at approximately 50 W, which included two 3-s sprints. Each athlete had a 3-min recovery period before the test was administered. All sprints were initiated from a stationary position and were divided by passive recovery.

The maximal power and work values were determined based on the best 6-s interval. Both absolute and relative values of work and power were calculated (kJ or kJ/kg and W or W/kg respectively). Additionally, the fatigue index, expressed as the percentage of decrement over repeated efforts, was calculated using the equations described by Orysiak et al. with adaptation to six repetitions in RSA^17^.

### Reaction Time (RT)

**Figure 2:**
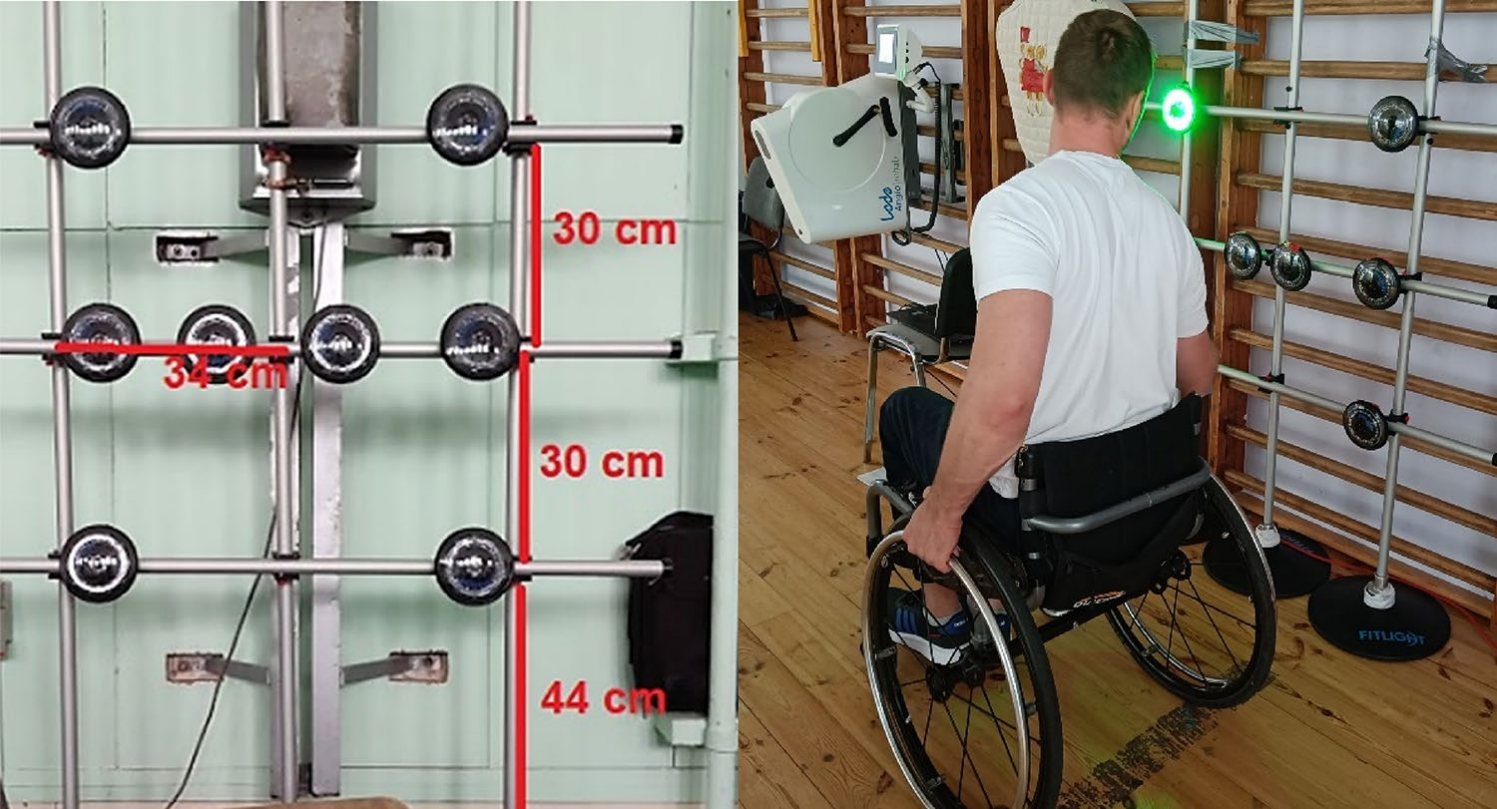
Reaction time measuring set-up. Left picture—lights setup, Right picture—participant setup.

The test was performed using the FitLight Trainer (FT) system (FITLIGHT Sport Corp., Ontario, Canada), which consists of a set of 8 lights with dedicated software arranged as shown in Fig. 2. The test was conducted in two trials—before and immediately after the maximal exercise test.

Prior to the measurement, participants performed a standardized tutored warm-up that included a practice reaction time test. The participants were then asked to assume a seated position in a wheelchair or chair with a backrest at such a distance from the lights that they were able to freely reach each of the LED lights with their working hand (without having to lean over). When the chair/carriage was set at the correct distance, this distance was marked so that in the postexercise test, the athlete could immediately be placed in the same position and distance from the measuring set. This was followed by a 60-s reaction time measurement using Fitlight. During the trial, participants were expected to respond to light signals (lights coming on). A dedicated program from the FitLight Trainer was used for recording the mean reaction time from the 60-s trial. During the test, reaction times were recorded before (RT1) and after (RT2) the trial. In addition, the difference in reaction time (ΔRT) under effort, calculated from the RT2-RT1 formula, was analyzed (negative values indicated an improvement in reaction time). The measuring position is shown in Fig. 2.

### Statistical analysis

Statistical analysis was performed using STATISTICA v. 13.0 (StatSoft). The normality of the data distribution was assessed using the Shapiro–Wilk test. Spearman's rank correlations were performed to assess the relationships between reaction times and the mechanical parameters. Paired t-test was used to assess differences between means in RT1 and RT2, but the Wilcoxon test was used in women group. T-test was used to reveal differences in RSA parameters between men and women. According to Cohen, the effect size for correlation is low if the value of r varies approximately 0.1, medium if r varies approximately 0.3, and large if r varies more than 0.5, and for differences in means when Cohen’s—d or Hedge’s—g varies approximately 0.2, medium if varies approximately 0.5, and large if varies more than 0.8^18,19^. A significance level of p < 0.05 was set.

## Results

**Figure 3:**
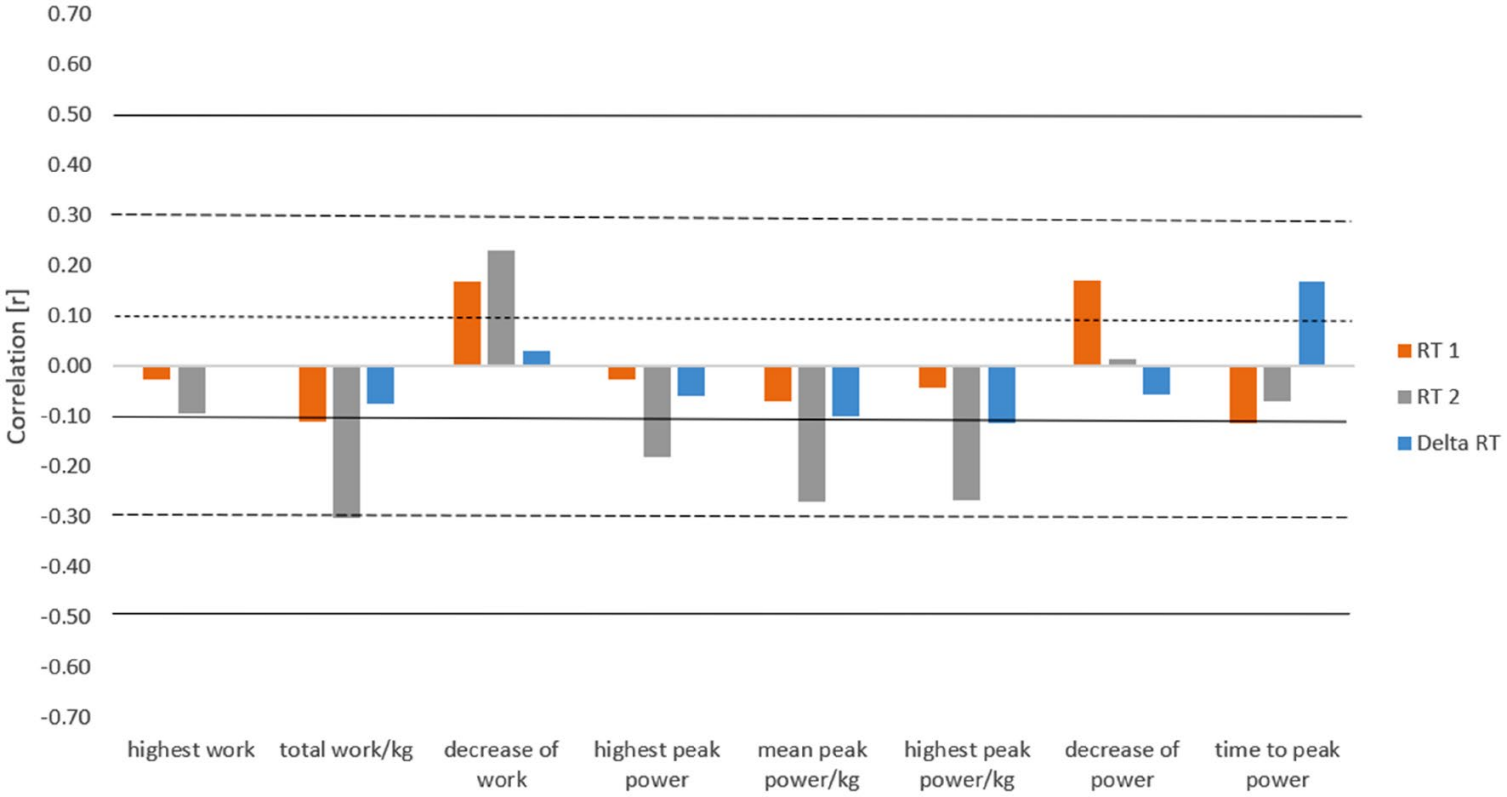
Correlation of reaction time (RT) before and after the RSA test with repeated sprint ability parameters (n = 18). RT1—reaction time before RSA; RT2—reaction time after RSA; ΔRT—delta of reaction time before and after RSA, dotted line—small correlation, dashed line—medium correlation, solid line—large correlation.

Statistical analysis revealed a moderate negative correlation between RT2 and total work, the highest peak power, the mean peak power, and the highest peak power/kg, and positive with a decrease of work (DW), but these relationships were not statistically significant (*p* > 0.05). Additionally, the correlation between RT1 or the delta of reaction time (ΔRT) and the mechanical parameters of the RSA test in the investigated group was not significant. The exact values of all the correlations can be found in Fig. 3.

Although there were large negative correlations between RT2 and decrease of power (DP) and between RT2 and time to peak power in females and positive between ΔRT and time to peak power in males (r > 0.5), these results were not statistically significant also (p > 0.05) (Tab. 1).

**Table 1:** Correlation between reaction times and anaerobic work capacity parameters. RT1- reaction time before RSA; RT2- reaction time after RSA; ΔRT—delta of reaction time before and after RSA; DW- decrease of work; DP- decrease of power.

	Highest work	Total work (kg)	DW	Highest peak power	Mean peak power (kg)	Highest peak power (kg)	DP	Time to peak power
M (n = 12)								
RT1								
* r*	− 0.077	0.007	0.280	− 0.063	0.028	0.056	0.371	− 0.014
* p*	0.81	0.98	0.38	0.85	0.93	0.86	0.24	0.97
RT2								
* r*	− 0.242	− 0.312	0.347	− 0.385	− 0.224	− 0.305	0.147	0.168
* p*	0.45	0.32	0.27	0.22	0.48	0.33	0.65	0.60
ΔRT								
* r*	− 0.266	− 0.326	− 0.336	− 0.319	− 0.308	− 0.301	− 0.056	0.560
* p*	0.40	0.30	0.29	0.33	0.33	0.34	0.86	0.06
F (n = 6)								
RT1								
* r*	0.314	− 0.143	− 0.314	− 0.143	− 0.086	− 0.086	− 0.371	− 0.314
* p*	0.54	0.79	0.54	0.79	0.87	0.87	0.47	0.54
RT2								
* r*	0.371	− 0.257	− 0.143	− 0.086	− 0.143	− 0.143	− 0.543	− 0.543
* p*	0.46	0.62	0.78	0.87	0.78	0.78	0.27	0.27
ΔRT								
* r*	0.348	− 0.029	0.290	− 0.174	− 0.174	− 0.174	− 0.406	− 0.116
* p*	0.50	0.96	0.58	0.74	0.74	0.74	0.42	0.83

**Figure 4:**
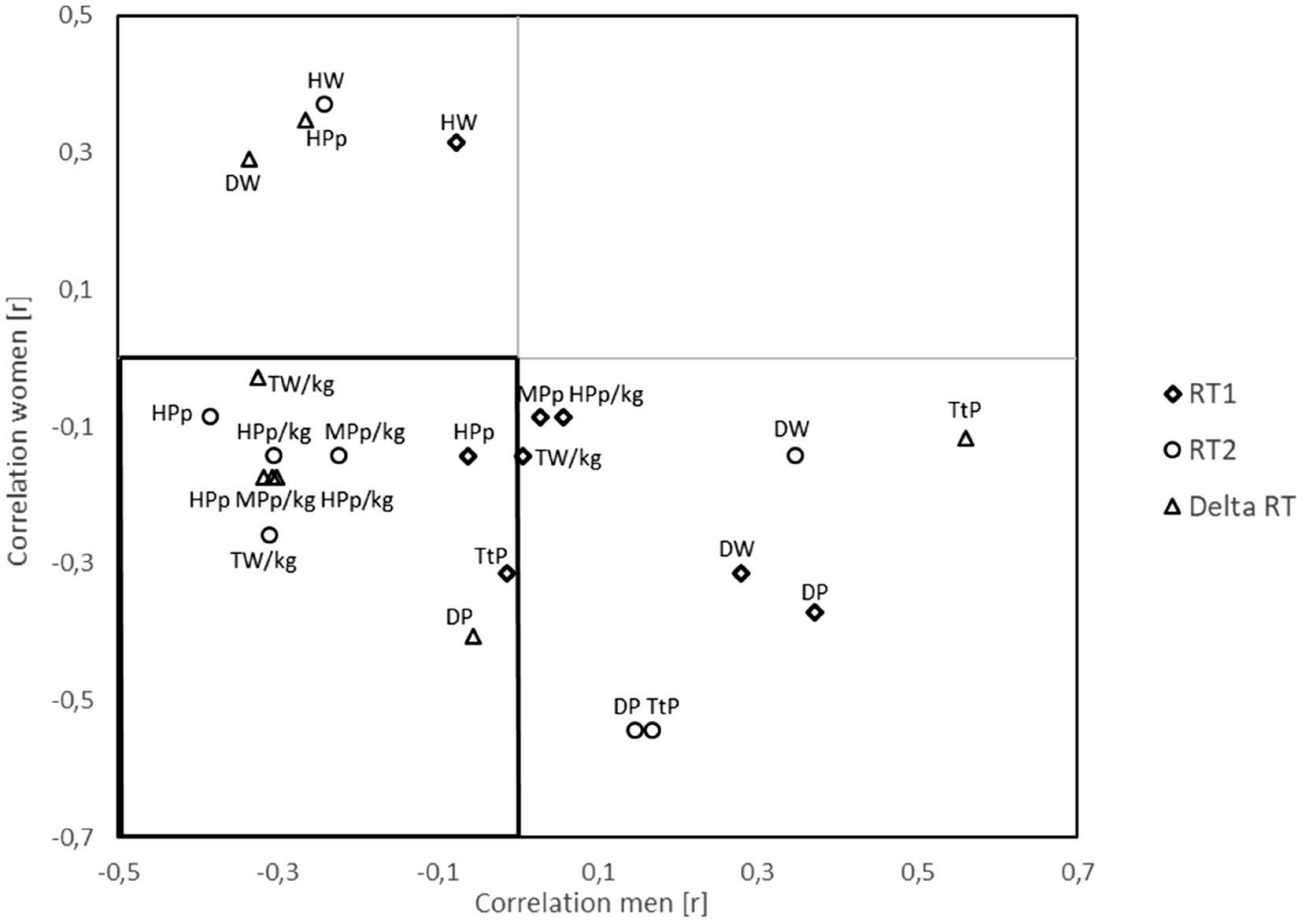
Scatter plot of the reaction time indicators and repeated sprint ability parameters correlations in men and women wheelchair fencers. RT1—reaction time before RSA; RT2—reaction time after RSA; ΔRT—delta of reaction time before and after RSA, DW—decrease of work, DP—decrease of power, HW—highest work, HPp—highest peak power; MPp—mean peak power TW—total work, TtP—time to peak power.

Moreover, the previously mentioned correlation results in Table 1, the scatter plot of correlation results for men and women, is shown in Fig. 4. A comparison of the correlation results between male and female athletes revealed five variables correlated with RT2, four with delta RT and two with RT1 having the same direction of the relationship in both sexes (Fig. 4).

**Figure 5:**
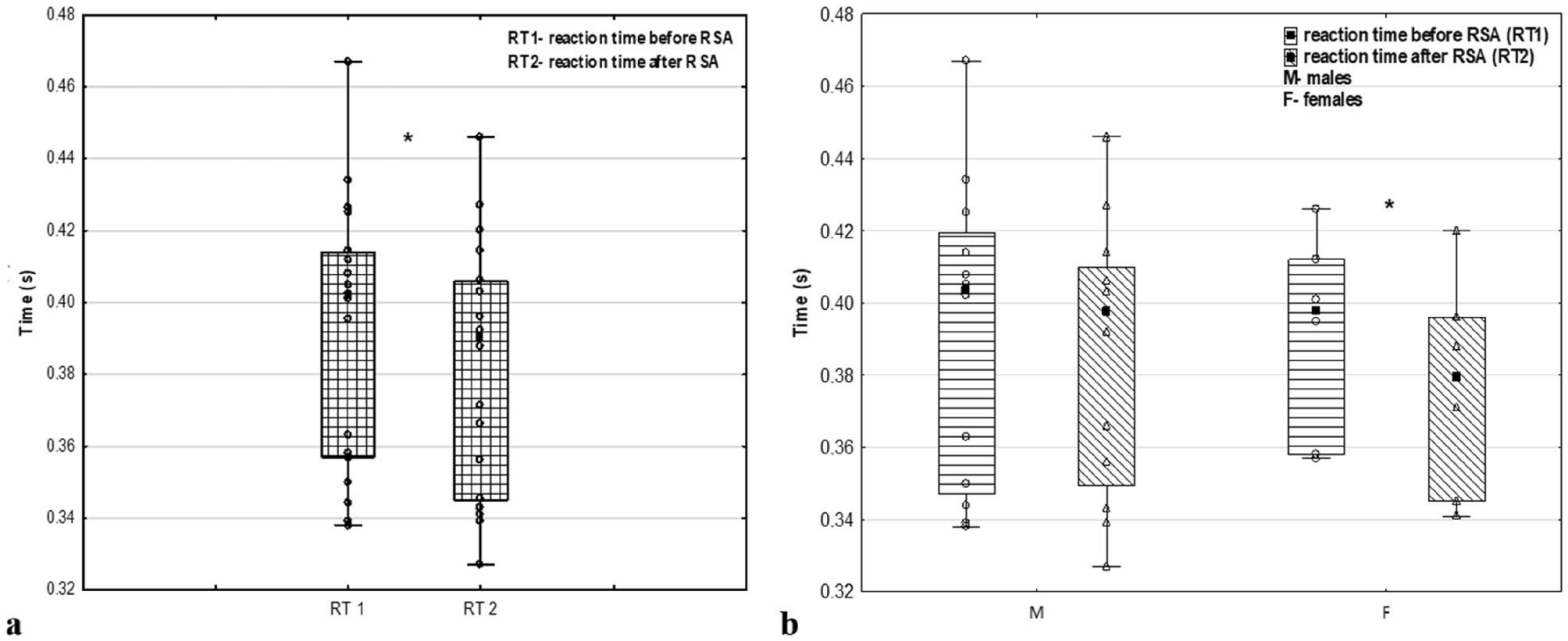
Reaction time values before and after the RSA test. RT1—reaction time before RSA; RT2—reaction time after RSA; M—males; F—females; *p ≤ 0.05; (**a**) comparison in whole group n = 18; (**b**) comparison according to sex M (n = 12) and F (n = 6).

The impact of the RSA test on RT was also assessed. The whole group of fencers achieved a significantly shorter average RT2 (p = 0.035; *d* = 0.54) after the RSA test (0.383 ± 0.035 s) than before the RSA test (0.391 ± 0.038 s). Additionally, RT2 was significantly shorter (0.377 ± 0.031 s) than RT1 (0.392 ± 0.028 s) in the women's group (p = 0.009; *d* = 1.69) (Fig. 5).

Moreover, males had significantly greater values of highest work, DW and highest peak power (p < 0.05) than females did. A statistical analysis also revealed a significantly greater total work value [J] in men than in women (p < 0.05). The detailed results from the above analysis are shown in Table 2.

**Table 2:** Differences in mechanical parameters between males and females.

	Males (n = 12)	Females (n = 6)	p-value	Hodge’s g
Total work				
[J]	10,853 ± 3846	7036 ± 1001	0.032*	1.31
[J/kg]	135 ± 65.6	111 ± 22.9	0.139	0.42
Highest work				
[J]	2122 ± 765	1278 ± 173	0.018*	1.31
[J/kg]	26.4 ± 9.00	20.1 ± 4.27	0.132	0.79
DW				
[%]	14.6 ± 4.78	8.32 ± 2.30	0.008*	1.51
Highest peak power				
[W]	430 ± 144	253 ± 31.9	0.009*	1.46
Mean peak power				
[W/kg]	4.82 ± 1.37	3.76 ± 0.61	0.095	0.89
DP				
[%]	26.5 ± 9.37	19.7 ± 8.42	0.154	0.75

DW—decrease of work; DP—decrease of power; *- p < 0.05.

## Discussion

In our work, we have examined the association between changes in RT before and after high-intensity repeated efforts and power and fatigue obtained in the RSA test in the national wheelchair fencer team. To the best of our knowledge, this is the first study assessing the relationship between speed-performance capacities determined by the test of repeated sprint ability and psychomotor ability in a WF group. The results indicate that the power, especially DP and time to peak power, in the RSA test is derived from the speed of movement, which is associated with a faster response to the stimulus. Furthermore, on this basis, it can be surmised which parameters are most closely related to the change in RT before and after high-intensity exercise. Analysis of the fencers without sex differentiation revealed a low or medium correlation between RT and mechanical parameters from the RSA test.

Considering sex, we found a high correlation between RT and RSA parameters. Interestingly, the power and fatigue obtained in the RSA test correlated with reaction time were not convergent between men and women. In between eleven parameters with the same direction of the relationship for men and women, only the highest peak power was (negatively) correlated with all cognitive measurements RT1, RT2 and delta RT. In our opinion, it can be a most diagnostic parameter to detect changes in RT and is not influenced by fatigue. The three other variables, relative highest peak power, relative mean power, and relative total work, are correlated (negatively) with postexercise cognitive function, described as RT2 and delta RT, which can correspond to fatigue. Therefore, we propose that these variables should be investigated in future research to identify versatile variables for both sexes. On the other hand, we can assume that male and female WF should be treated separately to monitor the impact of fatigue on RT.

The highest positive correlation was found between time to peak power and change in RT before and after the test. A decrease in the speed and power capacities described as a longer TtP, will be reflected in an increase in RT (Fig. 3). We assumed that this could be an effect of nervous system fatigue before the test. This negative impact of mental fatigue on reaction time was previously observed by Migliaccio et al.^20^. Central fatigue can also leads to a decrease in force production, which can be one of the explanations for our results^21–23^. The observed medium effect size of correlations between repeated sprint ability parameters and psychomotor indices with a lack of statistical significance suggested that the study should be continued on a larger group of WF athletes to confirm or deny our thesis.

In addition, the described analysis showed that there was an improvement in reaction time (RT) (shortening) after a series of repeated high-intensity arm-crank sprints, especially in the female group, even though men had faster reaction times than women did, which was also described by Lofti et al.^15^. This may indicate the positive effect of this type of exercise on neuromuscular stimulation in athletes and can be used as a part of warm-up during competitions. In research conducted on physically active men, authors observed an increase in cognitive function in simple tasks after high-intensity exercise^24^, which we also observed in our study (Fig. 5). On the other hand, high-intensity exercise impairs complex cognitive tasks^24^. Therefore, precise warm-up intensity prescription is necessary to avoid cognitive fatigue resulting in poor performance in WFs and decreased anaerobic performance, which was also observed in cyclists by Wittekind et al.^25^.

The RSA tests have been broadly studied in team sports assessing the impact of intermittent sprints on mechanical parameters based on running or cycloergometry tests^26,27^. In our work, we investigated sex differences during repeated high-intensity arm crank ergometry in wheelchair fencing. The men achieved higher absolute results for specific repeated sprint ability parameters than women. Similar to our results of sprint abilities were measured in abled-bodied team sport athletes^28^. Interestingly, unexpectedly in our study, there were no differences between men and women in terms of relative to body mass power or work values. We hypothesize that one of the limitations of these studies is the heterogeneity of disability among women and men, which, in our opinion, is one of the limitations of the study and should be considered in future analyses. Our second hypothesis is that in wheelchair fencing, strength and conditioning preparation are not essential for achieving good results, and most athletes are affected by their innate speed-strength abilities and is reflected in the RSA results.

## Application of the results

Incorporating the RSA test into pre-competition warm-up routines appears to hold potential for enhancing RT and overall athletic performance. The observed associations between indices of repeated sprint ability and RT pave the way for integrating specific exercises into training regimens. This approach conditions the body to efficiently regenerate phosphocreatine, which is essential for executing movements that require high power and speed. Furthermore, Malone et al.’s research indicates that improved RSA and speed metrics correlate with a greater ability to withstand higher workloads and a diminished risk of injuries among athletes. These findings warrant further exploration in the context of wheelchair fencing, where such adaptations could offer significant benefits^29^.

## Limitations of the study

Future research should encompass a broader cohort of fencers, with a particular emphasis on increasing female representation. The study was conducted on a small sample consisting of high-performance national team athletes with years of experience. This group of athletes went through a selection process through the years of training and competition, and in our opinion, their results are representative to draw a conclusion from the presented study.

Furthermore, it is recommended that participants be stratified according to a recognized scale, encompassing various levels of disability and sports classification in future research. Our study revealed differences in the relation between reaction time and mechanical parameters between male and female athletes. We assume that future research in more homogeneous groups stratified by type of disability or sport classification will result in a relationship between the RSA test and its impact on the changes in reaction time similar to those in division to gender, as presented in a recent study.

## Conclusions

Repeated high-intensity arm crank exercise has a positive impact on simple post-exercise cognitive tasks in wheelchair-treated patients, especially in women, and leads to a decrease in reaction time.

In the study several anaerobic mechanical parameters were established, that can be predictors of changes in reaction time in men and women wheelchair fencers.

## Data Availability

The data that support the findings of this study are not openly available due to reasons of sensitivity and are available from the corresponding author upon reasonable request.
